# How Can Sport-Based Interventions Improve Health among Women and Girls? A Scoping Review

**DOI:** 10.3390/ijerph20064818

**Published:** 2023-03-09

**Authors:** Maja Pedersen, Abby C. King

**Affiliations:** 1 Stanford Prevention Research Center, Stanford School of Medicine, Stanford University, Stanford, CA 94305, USA; 2 Department of Epidemiology and Population Health, and Stanford Prevention Research Center, Stanford School of Medicine, Stanford University, Stanford, CA 94305, USA

**Keywords:** review, health equity, gender equity, sport, women, physical activity, intervention, community-engaged research, participatory research, citizen science

## Abstract

Sport has been identified by the World Health Organization as an underutilized yet important contributor to global physical activity, by UNESCO as a fundamental right, and by the United Nations as a promising driver for gender equity through improved long-term health of women and girls. Although sport-based interventions have been popularized to advance educational, social, and political development globally, little attention has been given to its impacts on health outcomes among women and girls. We undertook a scoping review of research on sport-based interventions for health among women and girls to summarize current research approaches and findings. PRISMA scoping review guidelines were observed. Online databases (PubMed, PsycINFO, Web of Science) were used to identify peer-reviewed records published through August 2022. The interventions identified (*n* = 4) targeted health outcomes such as gender-based violence, HIV prevention, reproductive health, and child marriage. Based on our review, we recommend four key opportunities to advance the field of sport-based interventions in addressing health equity among women and girls. In addition, we highlight promising future research directions to broaden sport engagement of women and girls, improve long-term health, and build capacity toward health equity.

## 1. Introduction

Increasing physical activity (PA) is a global public health priority [1], given its well-established recognition as a protective factor in preventing and controlling noncommunicable diseases (NCDs) such as heart disease, stroke, diabetes, and several cancers [2]. Accordingly, the World Health Organization (WHO) has called for a “whole-of-society response to achieve a paradigm shift in both supporting and valuing all people being regularly active” (p. 6) [1], signaling a distinct focus on identifying and reducing disparities in PA participation. This effort is especially pertinent to groups impacted by health inequities, including women and girls.

Globally, PA rates of women are disproportionately lower than those of men, a phenomenon amplified among countries with overall lower amounts of activity [3,4]. PA participation differences are especially evident among youth. In recent decades, PA levels have increased among adolescent boys, yet have remained persistently low among girls, widening the gender gap and indicating distinct needs for PA promotion efforts customized to meet the needs and interests of girls [5]. One promising strategy for increased PA among women and girls is through sport. As defined by Khan and colleagues, sport is “a subset of exercise that can be undertaken individually or as a part of a team”, where “participants adhere to a common set of rules or expectations, and a defined goal exists” (pp. 59–60) [6]. Youth sport participation is associated with numerous positive outcomes, including improved physical and mental health, educational achievement, and social cohesion [7,8,9]. Such benefits may increase the likelihood that recommended levels of PA will be maintained into adulthood, generating beneficial impacts on health across the life span [7,10,11]. The benefits of sport participation are also evident in adults. Among untrained middle-aged adults, sport participation has been associated with reduced risk factors for NCDs and improved adherence to an active lifestyle, likely due to enhanced social interaction and quality of life [7,12,13,14].

Sport is recognized by the WHO as an underutilized contributor to global PA across all age groups [1], and the United Nations Economic, Social, and Cultural Organization (UNESCO) International Charter of Physical Education, Physical Activity and Sport identifies sport as a fundamental right for all and key to the realization of development and peace objectives [15]. The United Nations (UN) Entity for Gender Equality and the Empowerment of Women, UN Women, has emphasized the power of sport to address gender-based inequalities in and through sport, guided by the Sport for Generation Equality Framework [16]. Building on these global agendas, UNESCO’s Fit for Life initiative seeks to address the compounding crises of physical inactivity, mental health issues, social inequalities, and COVID-19 impacts among women and girls, with a key focus on multilevel efforts and inclusive sport policies to reduce physical inactivity and NCDs [17].

Sport for development (SFD) programs have been developed in response to the international calls to action and have been implemented widely [18]. The varied purposes of such programs, most of which originate in high-income countries [19], are generally organized around the UN’s mission to promote peace through sustainable development, and to improve the lives of people [20]. Research reports on SFDs have underscored the importance of engaging women and girls within these settings, and have openly identified the challenges and breakdowns of programs in achieving this end [21,22,23,24]. For example, a 2016 integrated literature review of SFD research and scholarship by Schulenkorf and colleagues, which included 437 peer-reviewed publications, identified a major gap in studies specifically dedicated to the advancement of access and rights for women and girls, and recommended future work to focus on gender equity [19]. Additional findings revealed predominant emphases by SFD interventions on social and educational outcomes (25% and 21% of publications, respectively), with markedly less research addressing health outcomes (13%). Furthermore, the analysis identified most studies as either conceptual (32%) or qualitative-only (38%). Despite these identified research gaps and myriad calls to action by prominent global health entities, a more recent review of the SFD literature, especially as it relates to gender equity and health-related outcomes among women and girls, is not present in the peer-reviewed literature.

Additional underexamined areas are practice-based ethical approaches and associated methodological sequelae. Sport for development programs have been criticized over aspects of power and politics, namely the use of “top-down” (versus “bottom-up”) development projects, which may emphasize foreign policy interests over lived realities of the women and girls they are meant to serve, and thus fail to effect real positive change [22,25]. Yet, while acknowledging these critical tensions, areas of hope for a more useful form of SFDs exist. Darnell and Hayhurst propose an intersection of decolonizing methodology and SFDs, where voices of the intended recipients of development are valued, reclaimed, and emphasized [26]. Parallel themes are evident in emergent forms of participatory research in the fields of public health and health promotion, which aim to reduce the impacts of power dynamics between researchers and non-researchers (e.g., participants, partnering entities, knowledge users) by actively engaging the groups and individuals in the research process who are most intimately connected to, and representative of, those intended to benefit [27,28].

In line with these goals, recent public health efforts attempting to establish “bottom up” approaches for action-oriented, transformative impact have paired community-engaged, participatory approaches with citizen science methodologies and technological advances to reveal the lived experiences of communities experiencing marginalization (including women and girls). These efforts seek to amplify the voices of marginalized groups as part of higher levels of decision-making and policy [29,30,31,32]. Such community-engaged participatory action approaches could help to address the identified needs within SFDs for improved understanding of local contextual factors influencing sport participation, including available resources, local partners, and socio-political context [33,34].

In summary, although sport has been widely identified as a potential driver of health equity among women and girls, the extent to which research studies have evaluated the impacts of such programming on health among these groups has not been explored. Likewise, the extent to which research studies have employed participatory research methods in this area is currently unknown. Therefore, the purpose of this investigation was to conduct a scoping review of sport-based interventions addressing gender equity issues among women and girls to (1) evaluate the impacts of these and other interventions on health-related outcomes, with an emphasis on PA, and (2) examine if and how participatory research approaches have been used. The overall aim of this English-language scoping review was to advance understanding of the current evidence base for sport-based interventions in relation to global public health goals, including increasing PA, among women and girls.

## 2. Materials and Methods

Scoping reviews are appropriate for synthesizing research evidence and mapping an existing body of literature in a given field in terms of nature and features [35]. Here, a scoping review was applied to summarize English-language research findings, examine how research is conducted in the field, identify research gaps, and generate ideas for future research [36]. The scoping review approach and reporting were guided by the PRISMA-ScR checklist [37]. The research questions were as follows:

1. What health-related outcomes have been evaluated within sport-based intervention research focused on gender equity among women and girls?
2. How have participatory research approaches been applied within sport-based intervention research focused on gender equity to enhance relevance and impacts among women and girls?

### 2.1. Inclusion and Exclusion Criteria

Inclusion and exclusion criteria were formed by the PCC mnemonic “Participants, Concept, Context” [35]. For Participants, we included all populations of women and girls, with no parameters on population characteristics (e.g., age, clinical factors, ability status, etc.) or form of gender identification (i.e., self-identified women and girls were categorized as such). For Concept, this review considered sport-based interventions using the description of sport provided by Khan and colleagues (i.e., a subset of exercise that can be undertaken individually or as a part of a team, where participants adhere to a common set of rules or expectations, and a defined goal exists) [6]. Also included were features provided by Elsborg and colleagues, in which sport-based recreation can take place in formalized organization and clubs or in more spontaneous, informal self-organized settings, with the purpose of participants performing the activity being primarily inherent in the activity [7]. As our purpose was to examine health-related outcomes, the study must have addressed or evaluated one or more health outcomes. Evaluation could be through quantitative, qualitative, mixed, or multiple methods. For Context, we included studies featuring a sport-based intervention according to the above definition (could be identified as SFD or not), and research conducted in a community setting, such as a school, community center, health center, or similar place. For types of studies, we limited study design to those evaluating the impacts of a program or intervention on individual or group outcomes, including experimental or quasi-experimental designs that used qualitative, quantitative, or mixed or multiple methods. No date range filter was selected, given that we could find no previous scoping or systematic reviews which had characterized the literature based on these parameters. The finalized inclusion and exclusion criteria are listed in Table 1.

### 2.2. Search Strategy

The search strategy was developed in consultation with an academic research librarian. Search terms were aligned with the inclusion criteria by using terms based on Participant/Concept/Context/Study type. The intentional use of “gender” rather than terms associated with “women” and “girls” allowed the search to include studies that might have incorporated both male and female participants but fit the inclusion criteria of more than 50% girls or women represented in the sample. An example PubMed search strategy is presented below (see Supplementary Material for details):

(“Sports”[MH] OR “Sports”[TW] AND (“Health Inequities”[MH] OR “Health Inequities”[TW] OR “Health Inequity”[TW] OR “Health Equity”[TW] OR “Health Disparity”[TW] OR “Health Disparities”[TW] OR “Gender Equity”[MH] OR “Gender Equity”[TW]).

The following databases were searched: PubMed, Web of Science, and PsycInfo. We adapted the search syntax to enhance sensitivity for each separate database. For PubMed, the search strategy was adapted to use MeSH Terms and Text Words to include a search within words contained in the titles, abstracts, other abstract MeSH terms, MeSH subheadings, publication types, author keywords, and other terms. For PsycInfo, we used Thesaurus words to index terms of relevant publications. The final database search was undertaken on 5 August 2022. To ensure thoroughness of the search, a manual search was performed based on the reference lists of the included articles.

### 2.3. Evidence Screening and Selection

Study selection was pre-specified in the protocol and guided by the inclusion and exclusion criteria. All identified citations were extracted from the databases and uploaded into Microsoft Excel, and all duplicates were removed using the automated function. The first author (MP) screened all titles and abstracts. Potentially relevant sources were retrieved in full, and full texts were assessed in detail in accordance with the inclusion and exclusion rubric. Reasons for the exclusion of full-text sources were documented. In accordance with the aim of the scoping review method, which is to characterize and disseminate the existing evidence rather than to provide a concrete synthesis and answer a clinically meaningful question, an assessment of methodological limitations or risk of bias of the evidence was not performed [35].

### 2.4. Data Extraction and Analysis

Data were extracted from the evidence sources guided by the objectives and research questions. This was completed by two reviewers: the lead author (MP) and a trained research assistant. A draft charting table was developed, piloted, and refined using an iterative process with two articles prior to use to ensure that all necessary data would be documented appropriately. The finalized charting tables were applied at the data extraction stage to record key information of the source and relevant results, including information on participant demographics, study setting, study design, research approach (subsections of participatory research methods and/or community engagement processes), theoretical framework or conceptual background, intervention, health outcome/focus, data collection methods/tools, short- and long-term outcomes, and suggestions for future research directions. In keeping with scoping review methods, we used basic descriptive analyses of both quantitative and qualitative results to clearly represent the sources of evidence.

## 3. Results

### 3.1. Selection of Evidence

**Figure 1:**
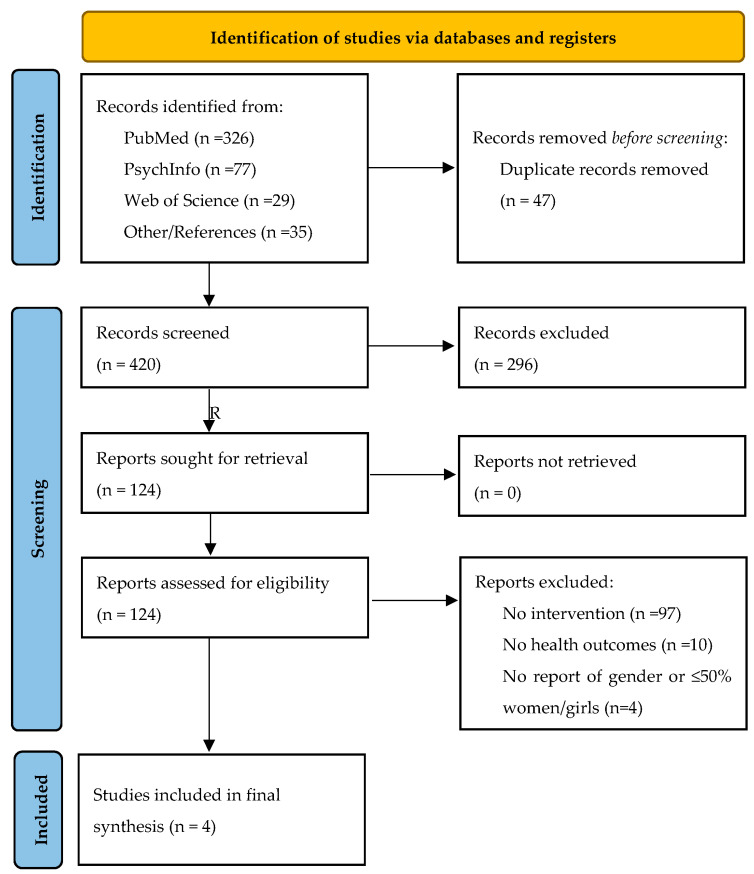
PRISMA flow diagram for scoping review.

Based on the database searches, 432 records were identified, and 35 records were identified through citation searching. After removing duplicates, 420 records were screened by title and/or abstract for relevance. Four studies met all eligibility criteria and were included in the final analysis. An overview of the evidence selection process is shown in the PRISMA flow diagram (Figure 1) [37].

### 3.2. Characteristics of the Evidence

The characteristics of the included studies are summarized in Table 2. Interventions (*n* = 4) addressed health outcomes among adolescent girls such as gender-based violence, HIV prevention, reproductive health, and child marriage. Qualitative and mixed method designs were used, and one study leveraged short message service (SMS) technology to improve participant engagement. The four evidence sources were linked to two primary projects: (1) *Parivartan for Girls* in India, where two evidence sources described results from one intervention study [38,39]; and (2) *SKILLZ Street* in South Africa, where one evidence source described intervention development and a pilot study [40], and another evidence source described a subsequent intervention study [41]. Below, the results are briefly described according to the PCC mnemonic [35], evaluation and results, and participatory and community-engaged approaches.

#### 3.2.1. Participants

Participants were adolescent girls, ages 11–17 years, and all programming was delivered by near-peer “mentors” or “coaches”, who were young women (ages 18–26) from the communities where the research took place. In the *Parivartan for Girls* studies, although demographics data were limited, the data reported characterized the participants (*n* = 15) as on average 15 years old (range 12–17 years), and primarily Muslim (90%) and Hindu (10%) [38]. For the *SKILLZ Street* studies in South Africa, demographic data were likewise limited. Reporting from the second intervention period characterized the participants (*n* = 382) as on average 11.9 years of age (range 11–16 years) and 82% black, with 47% speaking isiZulu at home [42]. Further information from the same study described results pertinent to the HIV prevention focus, including self-reported information on being in a partner relationship (33%), having ever had sex (7%), having ever experienced physical abuse (13%), and reporting emotional abuse (8%), and sexual abuse (16%) [41].

Although this scoping review included only studies reporting data on the recipients of the sport-based intervention, all four studies included additional perspectives collected via qualitative methods from intervention facilitators (mentors, coaches) and parents of the participants [38,39,40,41]. In one study, stakeholders such as school teachers and a social worker were involved in the research [41].

#### 3.2.2. Concept

The two interventions were developed based on male-only or mixed-gender interventions that preceded the single-sex versions. *Parivartan for Girls* was inspired by *Parivartan for Boys*, which was designed to promote gender-equitable attitudes among adolescent boys by having cricket coaches serve as role models for healthy masculinity [39]. *SKILLZ Street* was developed by Grassroots Soccer, a sport-based HIV prevention organization that had previously evaluated mixed-sex and male-only iterations [41].

The *Parivartan for Girls* studies used the sport of kabaddi as the foundation of the intervention. Kabaddi is a contact team sport popular in India, played between two teams of seven players on a court or field, and was selected based on its suitability for the context of limited equipment and space (e.g., no ball or equipment is required), and familiarity by community members [39]. The authors described the specific choice to not engage men and boys in the intervention as supporting the purpose of evaluating whether a “girl-centric” approach to change was possible in the setting. The strategies of change for this intervention were informed by theory (social norms theory, gender theory), formative research, and grounded knowledge of the community. The researchers posited that by participating in sport, girls would increase confidence and self-esteem, and by aspiring to a collective goal, the girls would learn negotiation skills and teamwork. The hypothesis was rooted in the idea that these skills would transfer to the girls’ individual lives and ultimately yield continued education and delayed marriage. The overall social and health outcome of focus was prevention of child marriage. The intervention was facilitated by trained female-only “mentors” and consisted of two components: (1) sport activities to improve physical fitness, and (2) participatory discussions on topics such as life skills, health, gender, violence, and sexual harassment to improve awareness and self-confidence. Additional emphasis was placed on family-based engagement strategies aimed at changing norms and beliefs among girls and their families, with the hypothesis that change at these levels might catalyze broader change in societal gender norms [39].

The *SKILLZ Street* studies used the sport of soccer as the foundation of intervention. Soccer was selected to enable female participants to challenge the common South African belief that soccer is a male-only sport [40]. The authors described the single-sex programming as a strategy to deliver gender-specific content and facilitate open conversation about sensitive topics on HIV and gender norms. The theory of change for this intervention was Social Learning Theory, which provided the rationale for engaging near-peer educators from the same community as facilitators or “coaches”. The 10-session intervention aimed to increase self-efficacy to avoid risky sexual behaviors, increase belief in gender-equitable norms, and facilitate access to and uptake of HIV counseling and testing services. The overall health focus outcomes were improved HIV prevention knowledge and attitudes, and beliefs and participation in HIV counseling and testing services. In the 2015 study, the intervention sessions were facilitated by trained coaches and consisted of (1) a life skills activity; (2) a soccer session where teams were paired to play a non-competitive soccer match; and (3) informal “team time”. Life skills topics were body image, sexual reproductive health and rights, HIV-related knowledge, and decision-making in relationships [40]. The 2018 study featured a revised curriculum, informed by the previous study, to integrate additional content on gender-based violence, and added a two-way messaging campaign component to complement curriculum learning and link participants with health services in their local communities [41].

#### 3.2.3. Context

The 15-month *Parivartan for Girls* intervention took place between December 2014 and March 2016 in the periphery of Shivaji Nagar, a large informal urban settlement in Mumbai, India. This area was described as containing predominantly Muslim migrant families from northern India, with substandard housing conditions, water supply, and sanitation. Social conditions were described as dominated by men, with girls’ mobility and visibility in public spaces restricted after menarche, and burkas or salwar kameez with a headscarf the dress code outside the home [38]. The studies grew out of a partnership between a grassroots non-governmental organization (NGO) with a longstanding presence in the areas, named Apnalaya, and the International Center for Research on Women, which was part of an international research consortium [38].

The initial 6-week *SKILLS Street* intervention study took place from January 2011 to December 2012 across five school-based sites in peri-urban areas of four provinces in South Africa: Alexandra, Khayelitsha, Kimberley, Port Elizabeth, and Soweto [40]. The subsequent 5-week *SKILLS Street* intervention study took place August–December 2013 at three primary school sites in the Soweto province, which is described as home to 1.69 million people, mostly of black African descent [41]. Grassroots Soccer, an established organization that implemented sport-based HIV prevention programming in schools across sub-Saharan Africa, developed the single-sex, girls-only curriculum, which was applied and tested in the two studies [40].

#### 3.2.4. Evaluation and Results

The *Parivartan for Girls* studies used a prospective design and qualitative methods, drawing on longitudinal interviews with athletes and mentors and detailed ethnographic field observations taken by research assistants. Thematic and narrative analysis methods were applied to the qualitative data. Findings from interviews with participants and mentors indicated the feasibility of an intervention combining sport with a health and gender-focused curriculum and emphasized the importance of visibility of girls participating in sports by their families and broader community in the process of social change. Mentor-based approaches were identified as effective in building local role models for adolescent girls, and engagement of mothers and families was also identified as important for facilitating change in the homes of participants [39]. Overall, based on qualitative evidence, the authors concluded that the *Parivartan for Girls* program changed what community members believed was acceptable about girls’ participation in sport and in public spaces [38].

The *SKILLZ Street* pilot study used mixed methods, including attendance tracking, HIV testing tracking, a pre–post survey on HIV-related knowledge and gender-equitable norms, and qualitative post-test focus groups with participants and coaches. Results included an 86.3% attendance rate, a 68.5% HIV testing rate, and strong evidence of improvement on 14 of the 16 survey items relating to HIV-related knowledge, attitudes, and communication. Qualitative evidence indicated overall positive responses from participants, including feelings of empowerment from playing soccer, importance of the female-only safe space, positive attitude change in gender norms, increased confidence and preparedness for challenges, and improved communication among peers and family about HIV-related topics [40].

The subsequent *SKILLS Street* intervention used a convergent parallel mixed methods design, where both quantitative and qualitative data were collected and analyzed separately, then integrated during the interpretation stage to consider convergence, divergence, contradictions, and relationship between methods [43]. Quantitative data were attendance tracking (but not tracking of HIV testing, due to controversial and challenging conditions) and a pre–post survey. Qualitative data collection was based on pre- and post-test focus groups and interviews with an emphasis on barriers and facilitators to program participation, and structured observation by study staff. Additionally, this study tracked SMS platform usage to gauge level of, and topics for, participant engagement. Program attendance was 97%, and survey results indicated a small-to-moderate improvement post-test. Qualitative results identified the sport of soccer and the coach-participant relationship as key motivators for participation, with barriers to participation oriented around conflicting duties and expectations at home, and more broadly community-level challenges (e.g., lack of basic resources at schools, scarcity of social/family support, prevalence of drugs and lack of police, tendency for adolescent girls to drop out of school). For the SMS evaluation, 69% of the participants accessed the SMS platform, and there were 160 requests for information about access to reproductive health services across the intervention period [41].

#### 3.2.5. Participatory and Community-Engaged Approaches

Both research programs used participatory and community-engaged approaches to intervention development and implementation. Partnerships between university-based research entities and NGOs (Apnalaya, Grassroots Soccer) formed local infrastructure supporting the research.

The *Parivartan for Girls* intervention used a series of participatory strategies at the design stage to operationalize intervention components and evaluate assumptions. For example, preceding intervention development, problem tree visualizations and safety mapping were employed with groups of girls, mothers, and fathers to explore local beliefs and identify potential concerns. A series of discussions engaged parents, girls, and community stakeholders in the selection and adaptation of kabaddi as the sport component of the intervention [39]. The partnership recruited and trained young women (ages 20–24) from within the informal settlement as mentors for the athletes. Mentors received training during a five-day residential instruction period that featured reflective sessions on gender equality, gender norms, and power relations. Mentors also participated in multiple in-depth refresher events focused on the curriculum and took part in monthly meetings with the NGO staff to plan forthcoming activities, discuss challenges, and identify best practices and solutions. The intervention curriculum emphasized participatory discussions between mentors and groups of athletes on a variety of topics designed to increase self-confidence, while parent reflection groups were convened to engage the families of the girls. A community advisory board was organized to enhance buy-in, as well as gain advice and feedback on all proposed activities. An additional component included two public tournaments that were intended to make visible the participation of athletes and mentors in kabaddi, challenging traditional norms of reduced or modest female mobility. These events were described as opportunities to disseminate intervention progress to families and community stakeholders [38].

The *SKILLZ Street* intervention was developed by NGO Grassroots Soccer in response to the disproportionate impact of HIV on young women, and was developed using an iterative process, described as an “evolutionary model of change” across progressively refined pilot efforts [40]. Multilevel stakeholder and community-based site engagement was described through approval by the South African Ministry of Education and partnership with school sites for recruitment and parent meetings [41], partnership with local HIV counseling and testing service providers [40], and partnership with a local healthcare center, including a session featuring a trained social worker representative as a guest speaker [41]. The *SKILLZ Street* intervention facilitators (coaches) were young women (ages 18–26 years) recruited in the local communities. Coaches were provided over 80 h of training through Grassroots Soccer, including knowledge-building on intervention content, facilitation techniques, and skills in working with youth [40]. In the second iteration of the study, coaches provided an optional additional 12 h of training on the SMS-based engagement program [41].

## 4. Discussion

The purpose of this scoping review was to describe the evidence of sport-based interventions addressing gender equity among women and girls with an emphasis on health-related outcomes, and to understand the extent to which participatory approaches have been applied. The four studies included in this review present important information concerning the potential impacts of sport-based interventions on health equity among women and girls. Although evidence sources were found to be few, the findings suggested individual and social-level changes in perceptions of gender norms and some health-related outcomes (e.g., improved individual and family-based efforts to resist harmful gender norms despite community pressure, improved individual knowledge, attitudes and beliefs about HIV-related topics). Notably, this review reveals that none of the studies directly evaluated the impact of sport participation on PA or PA-related behavior among participants. Furthermore, studies thus far have focused solely on adolescent girls. Although all studies included adult women as mentors or coaches, adults were not featured as recipients of sport-based interventions. Finally, this review underscored the relevance of community-engaged strategies to leverage local resources and partnerships across the included studies, and participatory strategies to engage multilevel stakeholders for transformative change.

### 4.1. Implications for Research and Practice

Based on the existing evidence and available literature, we provide the following recommendations to advance the field of sport-based interventions in addressing health equity among women and girls.

#### 4.1.1. Develop the Field’s Focus to Include Additional Health and Physical Activity Outcomes

Despite recognizing the use of sport as a driver for improved health, this review revealed a limited scope of current evidence on the impact of sport-based interventions on health outcomes. For example, none of the studies included in the review examined how sports-based interventions might contribute to improvements in PA behavior and engagement, whether within or outside of a sport context.

Sport-based interventions have been posited as highly relevant to public health, especially among vulnerable populations [7]. Improved understanding of how gender equity-focused sport-based interventions impact PA, sport engagement, and PA-related physical and mental health outcomes in the short- and longer-term across age groups can advance the field and may broaden the appeal of sports participation as a strategy to promote health equity through alignment with global health goals pertaining to noncommunicable disease and quality of life among women and girls [1,17].

#### 4.1.2. Incorporate Multilevel Approaches and Inclusive Practices to Uncover Structural Barriers and Supports to Sport Participation across the Life Course

Multilevel perspectives, which are rooted in ecological models of behavior [42], consider factors across individual, social, and environmental levels of behavioral influence, and are recognized as increasingly relevant to the advancement of equity-focused public health efforts [44]. Studies included in this review describe complex social and environmental conditions that impact development, implementation, and evaluation of sport-based interventions, and influence participation among athletes and mentors/coaches; however, outcomes were measured at the individual and social levels only. A recent systematic review of factors associated with sport participation among adolescent females identified relevant factors across biological, personal, social, socioeconomic, and environmental or resource levels [45], indicating the need for additional multilevel research approaches to provide a more complete picture of the field. Furthermore, application of theoretical models addressing broad levels of influence, such as policy, and incorporating targeted strategies across the life course, such as the Physical Activity and Sport Participation Framework which frames sport within the context of policies and approaches to achieve population-wide recommended levels of PA, can advance the field by linking evidence to actionable outcomes relevant across programmatic settings at local and national levels [46].

Future studies applying more focused and systematic strategies to document existing multilevel factors within the local context of participants’ daily lives can form an important foundation for developing intervention strategies to address barriers and leverage support. Such efforts may be particularly suited to include additional critical perspectives currently overlooked in this field, with emphases on emergent areas such as intersectionality (i.e., how overlapping social identities may compound experiences of discrimination within sport) [47], engagement of girls and women beyond heteronormative conceptions (e.g., inclusion of lesbian, gay, bisexual and transgender experiences) [48], and approaches customized for displaced women and girls [49]. Additional research is also needed to reveal experiences and perspectives of sport participation by women across the life course [50].

#### 4.1.3. Expand Community-Engaged and Participatory Research Approaches

Across studies included in this review, community-engaged and participatory research strategies were evident, but limited to the early stages of research. From a community engagement standpoint, all primary projects were developed and conducted through partnerships between one or more academic/research entities and a local or regional NGO with an existing relationship to the communities. Although stakeholders were identified across all studies, descriptions of power dynamics between partners and/or strategies for shared decision making, which are inherent principles within community-engaged scholarship, were not present [28]. To advance the field and provide benchmarks for ethical sport-based interventions focused on gender equity, future research design and reporting can better attend to foundational efforts in challenging hierarchies and control by systematically identifying and discussing these issues as part of the inner-workings of research and community relationships.

Likewise, participatory research approaches were applied across some, but not all stages of research. For example, the *Parivartan for Girls* project reported participatory approaches to study design (e.g., selection of sport, choice of setting for sport practice), and identified ‘participatory discussions’ in the intervention curriculum and strategies for family and stakeholder engagement, while the *SKILLZ Street* studies did not report or describe participatory practices. None of the studies reported participatory practices at stages of data analysis, interpretation, or dissemination.

Therefore, opportunities exist to better integrate participatory research practices into each stage of future research, from design through dissemination. These powerful practices are suggested by Darnell and Hayhurst as pathways forward in “prying open spaces to discuss the complex social problems addressed via Sport for Development and Peace research, while also exploring the diversity of knowledge at local, national, and global levels…” (p. 191) [26]. This approach can inform relevant considerations of sport-based intervention development and implementation in complex contexts such as rural and remote geographic locations, challenging weather and transportation conditions, areas of social conflict and violence, and program delivery amid rich linguistic and cultural diversity [34].

#### 4.1.4. Leverage Emerging Digital Technologies to Collect Data Relevant to Individual, Social, and Environmental Factors

The review identified one eligible study that used a digital health strategy (SMS) to enhance engagement and learning, and to improve awareness and links to local healthcare resources [41]. Accessibility was optimized in the two-way SMS campaign and reported to be well-received by participants and their families, with praise for enjoyment of learning via the mobile platform and the confidentiality it provided. These findings echo assertions of recent youth-focused health promotion research, which has emphasized the relevance and promise of digital tools for enhanced engagement, improved representation across diverse groups, and scale-up of research activities [29,51,52].

Digital health has been characterized as a strategy to bolster health equity among women, and access to such tools is increasing across low- and middle-income countries [53]. Progress may be achieved through pathways such as increased access to health care and services, empowerment gained by having access to one’s own health data, opportunities to capture relevant information, and improved visibility of issues such as gender-based violence, discrimination, and inaccessibility to health-bringing behaviors in their local environments [54,55,56,57].

### 4.2. Promising Directions: An Example Using the Our Voice Participatory Action Citizen Science Method

When paired with participatory research practices, digital technology can maximize engagement and amplify historically underrepresented voices, allowing researchers and communities to together gain insights on sport participation, health behavior, and gender equity. This can in turn pave the way for new approaches in practice- and policy-making spheres. One example of a research initiative toward this end is currently underway through a partnership between Peru-based NGO Fundación Deporte en Igualdad, Universidad Peruana de Ciencias Aplicadas, and USA-based Stanford University. The partnership links an established soccer-based intervention for girls and women with the *Our Voice* method, a participatory action citizen science research approach which features a multi-lingual app-based mobile platform for data collection and integration combined with facilitated multi-sectoral group discussions and action planning. These activities are aimed specifically at an improved understanding of diverse perspectives on local social and environmental health factors as well as formulating realistic solutions [58,59]. To date, female participants in urban areas of Peru have shared their findings directly with decision-makers representing schools, universities, municipal leadership, and representatives of the federal agency on sport. Continuing efforts are planned to facilitate relevant community-level actions based on information gained through this community-engaged multi-sectoral method, with future plans for project scale-up across age groups, residency status, and geographic settings. As in a number of *Our Voice* projects, outcomes are being measured at individual (e.g., physical and mental health), social (e.g., school and community program policies), and environmental (e.g., built features, safety) levels.

### 4.3. Strengths and Limitations

This scoping review has several strengths. First, the rigorous methodological framework used to explore existing literature allowed for a comprehensive picture of the current sport-based intervention literature using a gender-equity lens to evaluate impacts on health-related outcomes. Second, scholarship that moved beyond the conceptual and into action was highlighted, providing a state-of-the-science examples of action-oriented research that can contribute practical applications and essential knowledge to current global efforts. Third, evidence sources were evaluated for participatory practices and innovative strategies, delivering useful information that can be built upon in future global health research to promote gender equity through sport and PA.

This scoping review also has limitations. First, few articles were identified that matched our search criteria, creating a small evidence-source base. We opted to use a search strategy that focused on the concept of gender and gender equity, rather than on women and girls; this may have reduced the sensitivity of our search strategy to female-focused studies. Furthermore, it is highly likely that numerous sources exist in the gray literature and, additionally, that research on gender equity issues may not have explicitly used these terms in the publication narrative. However, analyzing the state of peer-reviewed evidence is critical to future scientific progress in this field of study. The topic of gender equity deserves increasing research focus to bring attention to critical issues across scientific disciplines, shed light on areas of need, and effect positive change. Second, although we included only full-text peer-reviewed publications, study quality was not specifically evaluated, which is characteristic of the scoping review methodology but may have impacted the findings of this review. Finally, no study included in this scoping review addressed the experiences and impacts of the COVID-19 pandemic. The devastating effects of the COVID pandemic on PA and sport participation, especially for women and girls, is currently being identified in the research literature [60], and future efforts, such as those aligned with UNESCO’s Fit for Life initiative, may be well suited to address the compounding effects of the COVID pandemic on gender equity in sport participation and PA engagement.

## 5. Conclusions

Current evidence on sport-based interventions to improve health among women and girls and address gender equity was described in what we believe to be a first-of-its-kind scoping review. The review found a dearth of current studies meeting basic eligibility criteria, indicating the critical need, and opportunities available, for advancing this research field. Based on the four qualitative and mixed methods studies found across two countries, we suggest the following to advance knowledge in this research area: (a) incorporate health and PA-related outcomes in research efforts involving sport-based interventions among women and girls; (b) emphasize multilevel approaches and inclusive strategies to engage underrepresented voices in the field of sport for gender equity; and (c) expand community-engaged participatory research to continue to grow a “bottom-up” evidence base through leveraging digital technologies and platforms to maximize opportunities for participation, inclusion, documentation, and information sharing and dissemination. Overall, this review highlights the promise of and need for expanded efforts in this area– efforts that are ethically attuned to the voices and perspectives of women and girls worldwide.

## Figures and Tables

**Table 1:** Inclusion and exclusion criteria.

Domain	Inclusion Criteria	Exclusion Criteria
Participants	Women and/or girls of all ages	Studies including ≤50% girls or women, studies not reporting gender distribution of sample Data from trainers, coaches, parents only
Concept	Sport-based interventions or programs per study definition Thematic focus on addressing gender equality and/or gender equity Thematic focus on improving health among participants Evaluation of one or more health outcome(s)	Physical activity interventions that encourage sport participation, but do not feature sport-based recreation or activities as a central component of the intervention protocol Thematic focus on improving performance of athletes Evaluation of social and/or educational outcomes only
Context	Sport for development programs and sport-based interventions Community settings	Intervention studies evaluating professional sport team performance or experiences
Type of studies	Intervention studies that use qualitative, quantitative, or mixed or multiple methods to evaluate the impacts of the intervention on participants Experimental or quasi-experimental designs Program evaluation studies including participant data Studies published in English	Observational designs Reviews Conference abstracts and editorials Conceptual studies Non-empirical studies and gray literature Studies published in languages other than English

**Table 2:** Characteristics of included studies.

Author, Year	Country	Health-Related Focus of Study	Participants	Intervention	Design, Method of Data Collection
Cislaghi (2020) [38]	India	Impact of kabaddi-based intervention (*Parivartan for Girls*) on gender-related social norms to prevent child marriage	Girls ages 12–16 years old (*n* = 15)	Duration of 15-months; Group-based, outside of school; facilitated by local, trained near-peer female mentors (20–24 years old); 2 sessions per week, 1.5 h each, including one session of life skills and gender training 1 session for playing kabaddi; Plus 2 public tournaments	Prospective qualitative research design, semi-structured interviews with participants only at two time points: 6 and 12 months; Field notes and observations
Collumbien (2019) [39]	India	Practice-based learnings and impact from the development and implementation of *Parivartan for Girls*	Same as above	Same as above	Longitudinal design, semi-structured interviews with participants (*n* = 15) and intervention mentors (*n* = 10) at three timepoints: 6 & 12 months, 1-year post-intervention; Field notes and observations
Hershow (2015) [40]	South Africa	Impact of soccer-based intervention (*SKILLZ Street*) to address three aims: (1) Increase self-efficacy to avoid risky sexual behavior; (2) Increase belief in gender equitable norms; (3) Facilitate access and uptake of HIV counseling and testing services	Girls ages 11–14 years old (*n* = 4260)	Duration of 6-weeks; Group-based, after school; facilitated by local, trained near-peer female coaches; 2 sessions per week, 2 h each, including a life skills activity, a non-competitive soccer match, and informal ‘team time’ facilitated by coaches	Mixed methods design. Quantitative data: program attendance, HIV testing rate, pre/post questionnaire (*n* = 514) on HIV-related knowledge, attitudes, and communication. Qualitative data: focus groups with participants (*n* = 11 groups) and coaches (*n* = 5 groups)
Merrill (2018) [41]	South Africa	Same as above, plus evaluation of SMS-based component and implementation factors	Girls ages 11–16 years old (*n* = 394)	Same as above, but delivered across 5-weeks, with intervention content revise to add focus on gender-based violence, and addition of a two-way SMS-campaign	Convergent parallel mixed-methods design. Quantitative data: program attendance, SMS campaign platform usage, pre/post questionnaire. Qualitative: focus groups with participants (*n* = 3 groups), parents of participants (*n* = 2 groups), coaches (*n* = 1 group); individual interviews (*n* = 4) with parents of participants and a social worker engaged in project. Structured observation and fidelity checks

## Data Availability

Not applicable.

## Supplementary Materials

The following supporting information can be downloaded at: https://www.mdpi.com/article/10.3390/ijerph20064818/s1, (1) PubMed Search Terms; (2) Preferred Reporting Items for Systematic reviews and Meta-Analyses extension for Scoping Reviews (PRISMA-ScR) Checklist.

## Abbreviations

NCDs	Non-communicable diseases
NGO	Non-governmental organization
PA	Physical activity
SFD	Sport for development
SMS	Short message service
UN	United Nations
UNESCO	United Nations Educational, Scientific, and Cultural Organization
WHO	World Health Organization
