# Supplementary material for: How Can Sport-Based Interventions Improve Health among Women and Girls? A Scoping Review
Source: Int J Environ Res Public Health. 2023 Mar 9;20(6):4818. doi: 10.3390/ijerph20064818 (PMC10049722; doi:10.3390/ijerph20064818)
Supplement: Supplementary file 1 [file ijerph-20-04818-s001.zip › Supp_Mat_PubMed Search Strategy Example.pdf]

PubMed Search MeSH Terms:

**MeSH Term**	**Sports**	**Health Equity**	**Health Inequities**	**Gender Equity**
MeSH ID #	D013177	D000069576	D000091682	D000084803
Entry Terms	Sport<br>Athletics<br>Athletic	Equity, Health	Health Inequity<br>Inequities, Health<br>Inequity, Health<br>Health Inequalities<br>Health Inequality<br>Inequalities, Health<br>Inequality, Health	Equity, Gender<br>Gender Equities<br>Gender Inequality<br>Gender Inequalities<br>Inequality, Gender<br>Gender Inequity<br>Gender Inequities<br>Inequity, Gender<br>Gender Equality<br>Equality, Gender<br>Gender Equalities
